# Inhibition of immune checkpoints prevents injury-induced heterotopic ossification

**DOI:** 10.1038/s41413-019-0074-7

**Published:** 2019-11-01

**Authors:** Chen Kan, Jiazhao Yang, Ding Na, Yuanhong Xu, Baixia Yang, Haodong Zhao, Huadong Lu, Yuyun Li, Keqin Zhang, Tammy L. McGuire, John A. Kessler, Lixin Kan

**Affiliations:** 1 School of Basic Medical Sciences, Anhui Medical University, 81 Meishan Road, 230032 Hefei, Anhui China; 2 Department of Traumatic Orthopedics, Anhui Provincial Hospital, The First Affiliated Hospital of China University of Science and Technology, 233000 Hefei, Anhui China; 3 Department of Clinical Laboratory, the First Affiliated Hospital, Anhui Medical University, Hefei, Anhui China; 4 Department of Emergency, the First Affiliated Hospital, Anhui Medical University, 230000 Hefei, Anhui China; 5 Emergency Medicine Center, Anhui Provincial Hospital, The First Affiliated Hospital of University of Science and Technology of China, Hefei, Anhui China; 6 Department of Medical Laboratory Science, Bengbu Medical College, 2600 Donghai Avenue, Longzihu, 233030 Bengbu, Anhui China; 7 Department of Endocrinology and Metabolism, Tongji Hospital, Tongji University School of Medicine, Shanghai, China; 8 Department of Neurology, Northwestern University, Ward Building 10-233, 303 East Chicago Avenue, Chicago, IL 60611-3008 USA

**Keywords:** Bone, Physiology

## Abstract

Heterotopic ossification (HO), true bone formation in soft tissue, is closely associated with abnormal injury/immune responses. We hypothesized that a key underlying mechanism of HO might be injury-induced dysregulation of immune checkpoint proteins (ICs). We found that the earliest stages of HO are characterized by enhanced infiltration of polarized macrophages into sites of minor injuries in an animal model of HO. The non-specific immune suppressants, Rapamycin and Ebselen, prevented HO providing evidence of the central role of the immune responses. We examined the expression pattern of ICs and found that they are dysregulated in HO lesions. More importantly, loss of function of inhibitory ICs (including PD1, PD-L1, and CD152) markedly inhibited HO, whereas loss of function of stimulatory ICs (including CD40L and OX-40L) facilitated HO. These findings suggest that IC inhibitors may provide a therapeutic approach to prevent or limit the extent of HO.

## Introduction

Heterotopic ossification (HO),^1–3^ acquired or hereditary, is characterized by pathological bone formation outside of the normal skeleton, generally following tissue damage. For example, acquired HO (aHO), is commonly triggered by traumatic brain injury, spinal cord injury,^4,5^ total hip arthroplasty,^6^ wartime trauma, or other traumatic injuries.^4,5,7^ Following the acute injury, these patients typically develop persistent low-grade inflammation, chronic pain, unhealed wounds, restricted joint movement, nerve entrapment, and diminished quality of life. Hereditary HO, such as fibrodysplasia ossificans progressiva (FOP),^8^ though rare, is much more devastating and life threatening. Notably, even though FOP is caused by gain-of-function mutation of the type 1 bone morphogenetic protein (BMP) receptor,^9,10^ ACVR1 (also known as ALK2), the initiation of the HO process in FOP is similarly triggered by abnormal immune responses to minor injuries (also called flare-up) followed by persistent low-grade inflammation.

To model HO in mice, we created a transgenic line (Nse-BMP4) that overexpresses BMP4 under the control of the neuron-specific enolase (Nse) promoter.^11,12^ Nse is an important glycolytic enzyme that is modulated by the cellular milieu in response to traumatic injury. In Nse-BMP4 mice, the Nse transgene is induced in macrophages (Mϕ) by injury, and the HO that develops in Nse-BMP4 mice is restricted to the site of the injury. The injury-induced local overexpression of BMP4 becomes significant only 3 days post injury (see Supplementary Fig. 1) indicating that upregulation of BMP signaling is not the initiating event in the subsequent signaling cascade that leads to HO.

In previous studies, we found that both innate immune responses and adaptive immunity play key roles in the pathological process of HO.^11,12^ Similarly, recent studies have also shown that disrupted adaptive immune responses are closely associated with HO formation in mice following burn injury and Achilles tenotomy.^13^ The overall breadth and magnitude of immune responses are regulated by immune checkpoint proteins (ICs).^14–16^ The mammalian genome encodes many different ICs with different expression patterns and functionalities, and some ICs stimulate immune responses to maintain immune homeostasis, whereas others are inhibitory. The central hypothesis of this study is that tissue damage, especially after traumatic injuries,^17–19^ induces IC dysregulation leading to a cascade of abnormal immune responses that culminate in HO. This suggests that correcting immune homeostasis using IC inhibitors could be a novel therapeutic approach for prevention and/or treatment of HO.

To test this hypothesis and to explore potential translational applications, we used non-specific immune suppressants including Rapamycin^20^ and Ebselen^21^ (1) to clarify the role of altered immune homeostasis in HO; (2) substantiated that there is local dysregulation of IC during the HO process; and (3) functionally tested the relationship between IC dysregulation and HO using neutralizing antibodies (Abs) against typical ICs.

Currently, there is no effective treatment for preventing or limiting the extent of HO. Our observations suggest that currently available, Food and Drug Administration (FDA)-approved IC inhibitors may potentially provide a practical therapeutic approach to the disorder.

## Results

### Evidence of altered immune homeostasis in an HO animal model

**Fig. 1:**
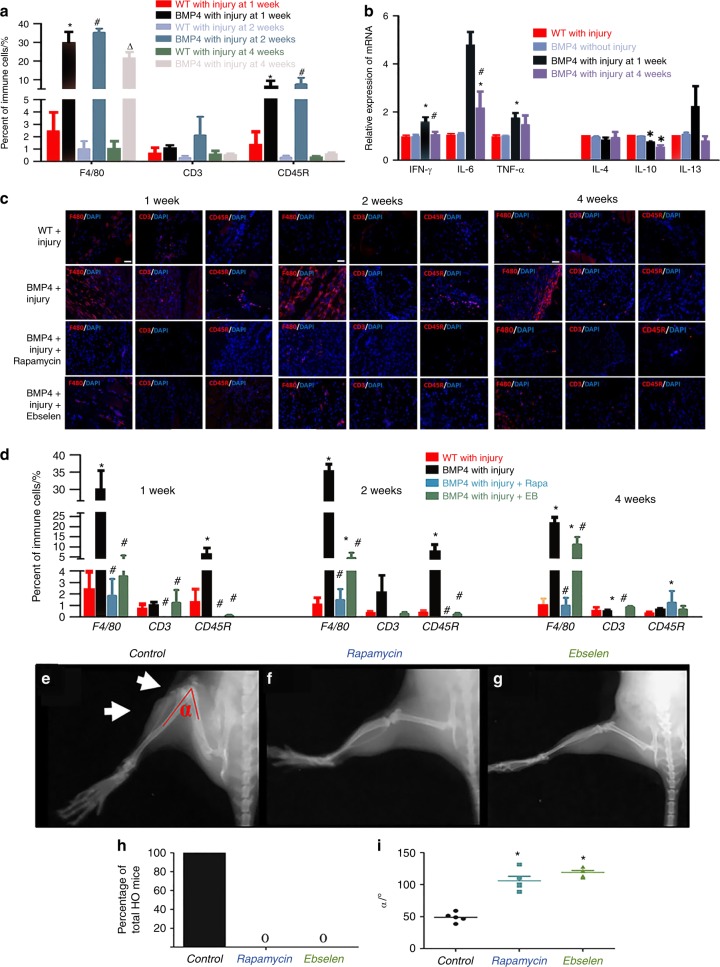
Evidence of altered immune homeostasis in an HO animal model. **a** Quantification of local infiltrating immune cells in local lesions in WT and Nse-BMP4 mice at different time points p.i. (*n* = 4 mice per group), *P < 0.05 vs group of WT mice with injury at 1 week p.i., ^#^*P* < 0.05 vs group of WT mice with injury at 2 weeks p.i., and △*P* < 0.05 vs group of WT mice with injury at 4 weeks p.i. **b** qRT-PCR results show the expression of pro-inflammatory cykokines (IFN-γ, IL-6, and TNF-α) and anti-inflammatory cytokines (IL-4, IL-10, and IL-13) in the lesions at different time points p.i. (*n* = 5 mice per group), *P < 0.05 vs group of WT mice without injury, ^#^*P* < 0.05 vs WT mice with injury. **c**, **d** Immunofluorescent images and quantification of Rapamycin- and Ebselen-mediated suppression of WBC infiltration into the lesions at 1, 2, and 4 weeks p.i. (*n* = 4 mice per group), *P < 0.05 vs group of WT mice with injury, ^#^*P* < 0.05 vs group of Nse-BMP4 with injury. **e**–**h** X-ray imaging revealed that both Rapamycin and Ebselen prevented HO and increased the range of joint motion (**i**) at the lesion site (*n* = 5 mice per group). White arrows point to HO and joint limitation in the control, *P < 0.05 vs control group. Statistics were performed using a repeated-measures ANOVA (**a**, **b**, **d**) or ANOVA (**i**) with Bonferroni’s post hoc test. Scale bar = 200 μm

The Nse-BMP4 transgenic mouse recapitulates the hallmarks of both aHO and FOP (refs. ^11,12,22–25^ and Supplementary Fig. 1). We examined the infiltration of immune cells into sites of minor injury and the production of cytokines in Nse-BMP4 and wild-type (WT) mice and in uninjured controls at different time points post-injury (p.i.). Infiltration into the injury sites of Mϕ/monocytes (F4/80), T cells (CD3), and B cells (CD45R) was examined by immunohistochemistry, and responses to the same injury differed significantly in Nse-BMP4 and WT mice (Fig. 1a, c). All tested immune cells were found in the HO lesions. The distribution patterns varied greatly for different subpopulations of cells at different time points, but Mϕ were the predominant infiltrating immune cells throughout the entire HO process. We also measured the expression of inflammatory cytokines in the lesion sites using quantitative real-time reverse transcription polymerase chain reaction (qRT-PCR) and found that all the tested cytokines were dysregulated. The pro-inflammatory cytokines, interferon (IFN)-γ and tumor necrosis factor (TNF)-α, were transiently increased at 1 week p.i., but were decreased at 4 weeks p.i. in Nse-BMP4 mice (Fig. 1b). In contrast, the opposite trend was observed for the anti-inflammatory cytokine, interleukin (IL)-10.

### Non-specific immune suppressors, Rapamycin and Ebselen, inhibit HO

We next tested the causal relationship between the altered immune responses and the subsequent HO. We repeated the injury procedure and then treated the mice with Rapamycin or Ebselen, for 2 weeks. Rapamycin and Ebselen each inhibited local immune cell infiltration (Fig. 1c, d). X-ray imaging revealed that Rapamycin and Ebselen also each inhibited HO formation (Fig. 1e–h) and increased the range of joint motion at the injury site (Fig. 1i). Rapamycin and Ebselen were effective in preventing HO when treatment was initiated any time during the first 10 days after the injury. Treatment started after the first 10 days no longer altered the progression of HO (Supplementary Fig. 2). Other than inhibition of HO formation, no effects on bone were noted for either Rapamycin or Ebselen either radiographically or histopathologically.

### Dysregulation of ICs in HO

**Fig. 2:**
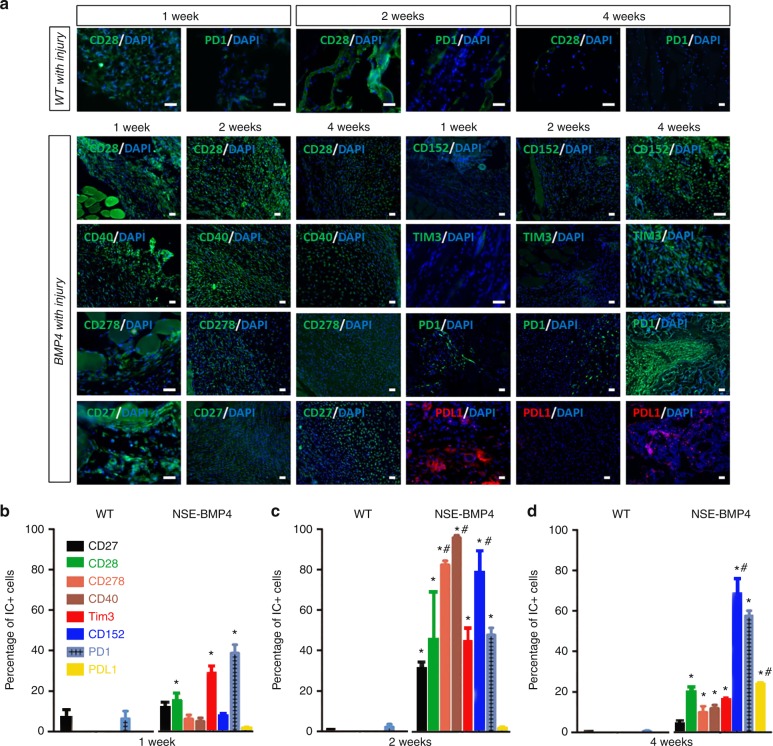
Immune checkpoint proteins are dynamically dysregulated in HO lesions. **a** Immunofluorescence staining showed the expression of CD28 and PD1 in WT mice at 1, 2 and 4 weeks p.i. In Nse-BMP4 mice, immunostaining revealed a distinct expression pattern of stimulatory ICs (CD27, CD28, CD40, CD278) in HO lesions at 1, 2, and 4 weeks p.i. **b**–**d** Quantitation of the percentages of cells expressing IC proteins in Wt and Nse-BMP4 mice at 1, 2, and 4 weeks p.i. (*n* = 4 per group per time point). In WT mice with injury, the percentages of cells expressing ICs were generally low. In contrast, in Nse-BMP4 mice, the stimulatory ICs, including CD27, CD28, CD278, and CD40, and inhibitory ICs, including Tim3, CD152, PD1, and PD-L1, were differentially dysregulated. Note that stimulatory ICs generally were abnormally increased at early stages and then decreased, while some inhibitory ICs remained increased at late stages. *P < 0.02 compared to wild-type control at all time points. ^#^*P* < 0.05 compared to 1 week time point in the Nse-BMP4 group. Statistics were performed using a repeated-measures ANOVA with Bonferroni’s post hoc test. Scale bar = 200 μm

**Fig. 3:**
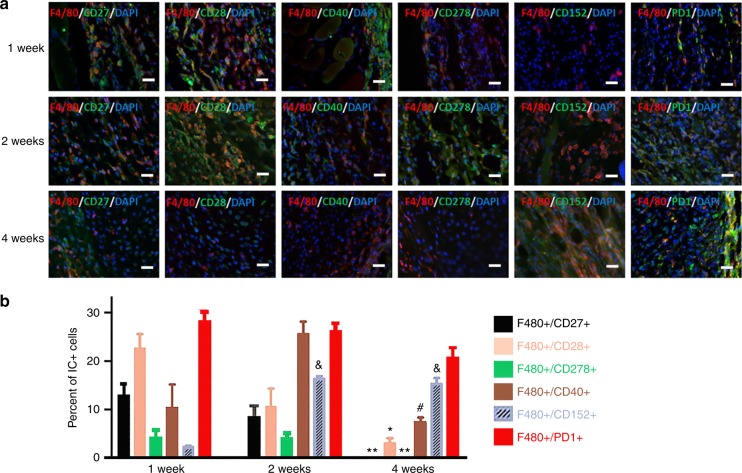
Immune checkpoint proteins were extensively co-localized within macrophages. **a** Immunostaining showed the co-localization of F4/80 and ICs at 1, 2, and 4 weeks p.i. **b** Double-staining of ICs with F4/80 found that, within the macrophage population, stimulatory and inhibitory IC+ cells followed similar respective patterns (*n* = 4). Scale bar in all panels = 200 μm *Differs from 1- and 2-week groups at *P* < 0.05; **Differs from 1- and 2-week groups at *P* < 0.01; ^&^Differs from 1-week group at *P* < 0.05; ^#^Differs from the 2-week group at *P* < 0.05. Statistics were performed using a repeated-measures ANOVA with Bonferroni’s post hoc test. Scale bar = 200 μm

To directly test the central hypothesis that tissue damage, especially after traumatic injuries, induces IC dysregulation in susceptible mice, we next determined whether ICs are dysregulated in the HO process. We immunostained injury sites for ICs including stimulatory ICs (CD27, CD28, CD278, CD40), and inhibitory ICs (CD152, TIM3, PD1, and PDL1) at different times p.i. (Fig. 2a–c). Although the ICs had somewhat differing patterns of expression, in general the percentage of cells expressing stimulatory ICs was markedly increased in the early stages but decreased at later stages of HO. The time course of expression of inhibitory ICs was more variable with increases in some (TIM3 and PD1) inhibitory ICs occurring within the first week, whereas the percentage of cells expressing other inhibitory ICs was increased later during the HO process. Double staining with immune cell markers (F4/80) revealed that both stimulatory and inhibitory ICs were extensively co-expressed by F4/80+ Mϕ (Fig. 3) but only rarely in lymphocytes (Supplementary Fig. 3).

### Neutralizing Abs against inhibitory ICs block HO

**Fig. 4:**
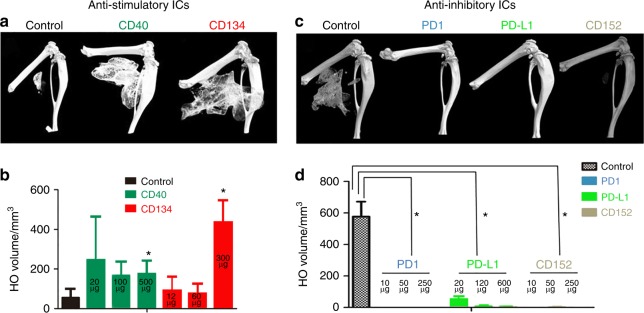
Loss of function of inhibitory ICs blocks HO, while loss of function of stimulatory ICs facilitates HO. **a**, **b** Neutralizing antibodies against stimulatory ICs (CD40 and CD134) facilitated HO formation (*n* = 3) *P < 0.05 vs control group. **c**, **d** Neutralizing antibody against inhibitory ICs (PD1, PD-L1, and CD152) almost completely inhibited HO formation (*n* = 3), *P < 0.05 vs control group

**Fig. 5:**
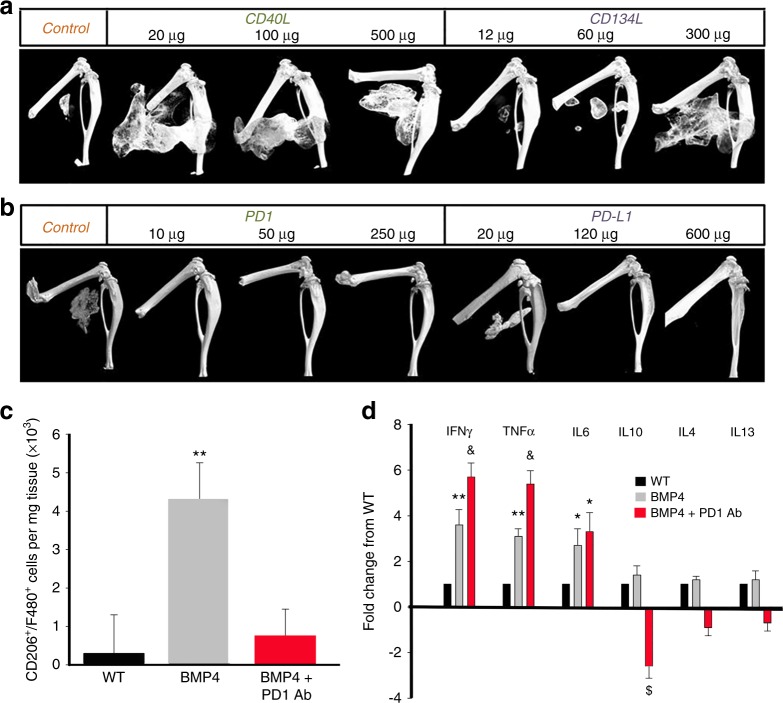
Dose response of the effects of IC inhibition on HO and effects of PD1 antibody on CD206+/F480+ macrophage numbers and cytokine expression. **a**, **b** The effects of IC antibodies, particularly CD134L and PD-L1, on HO are dose dependent. **c** Effects of PD1 antibody (10 μg) on the number of CD206+/F480+ cells in tissue. Values are means ± SEM. *N* = 5. **Differs from other groups at *P* < 0.01. **d** Cytokine mRNA expression in Nse-BMP mice with and without PD1 antibody. Values are expressed as fold-change from the WT level. *N* = 4. *Differs from WT at *P* < 0.05; **Differs from WT at *P* < 0.01; ^&^Differs from both other groups at *P* < 0.02; ^$^Differs from both other groups at *P* < 0.05. Statistics were performed with ANOVA with Bonferroni’s post hoc test

To functionally test the central hypothesis, we treated the mice with neutralizing Abs against stimulatory ICs (Fig. 4a, b) or inhibitory ICs **(**Fig. 4c, d) after injury. Typical three-dimensional (3D) reconstructed micro-computed tomographic (micro-CT) images shown in Fig. 4 demonstrate that neutralizing Abs against stimulatory ICs (CD40 and CD134) actually facilitated HO, while neutralizing Abs against inhibitory ICs (PD1, PD-L1, and CD152) inhibited HO, partly in a dose-dependent (Fig. 5) manner. Note that, in the studies shown in Fig. 4 of Abs against inhibitory ICs, most mice in the experimental groups did not develop HO at 1 month. To determine whether the treatments only delayed the HO process, the experiment was extended to 2 months. For this reason, the micro-CT images in Fig. 4c were taken at 2 months p.i., instead of 1 month p.i.(Fig. 4a). The HO volume difference between the control groups in (Fig. 4a) and (Fig. 4c) reflects this difference. The effects of neutralizing inhibitory ICs were profound with almost no mice developing HO even after 2 months (Fig. 4d). Neutralization of PD1 increased the expression of inflammatory cytokines (IFN-γ and TNF-α) in BMP4-induced Mϕ, decreased expression of anti-inflammatory cytokines (IL-10), and decreased the population of CD206+ Mϕ, suggesting polarization toward a more inflammatory phenotype (Fig. 5).

### HO is associated with excessive collagen deposition and with changes in metallopeptidase expression by Mϕ

**Fig. 6:**
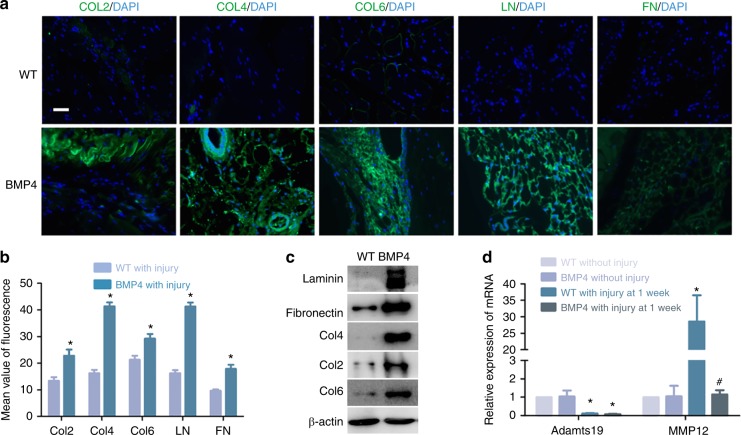
Increased deposition of ECM proteins in HO lesions is associated with decreased expression of Adamts19 and MMP12 deficiency in macrophages. **a**, **b** Immunostaining revealed that deposition of ECM proteins (collagen 2, 4, 6, laminin, and fibronectin) is increased in HO lesions. (*n* = 3 per group), *P < 0.05 vs group of WT with injury. **c** Western blot analysis confirms the increase in ECM proteins in injury sites in Nse-BMP4 mice. **d** qPCR results showed that Adamts19 and MMP12 expression was significantly decreased in F4/80 macrophages from Nse-BMP4 mice with injury (*n* = 3 per group), *P < 0.05 vs group of WT without injury, ^#^*P* < 0.05 vs group of WT with injury at 1 week p.i

We found that increased deposition of extracellular matrix (ECM) proteins is a consistent feature of the early phases of HO (Fig. 6a–c). In view of the central role of Mϕ in HO, we questioned whether they may alter the ECM during the HO process. Matrix metallopeptidase 12 (MMP12) and a metalloproteinase with thrombospondin motifs 19 (Adamts19) are important ECM components involved in the breakdown of ECM. Expression by F4/80 Mϕ of Adamts19 was reduced after injury in WT mice but MMP12 expression was significantly increased. However, the increase in MMP12 expression did not occur after injury to Nse-BMP4 mice (Fig. 6d), suggesting a possible mechanism for the increase in ECM proteins in the lesional tissues.

## Discussion

HO typically is preceded by trauma followed by an aggravated early acute injury response, and then persistent low-grade inflammation.^26^ However, the cascade of events leading to HO is still largely unknown, especially the early triggering event. The predominant view in the field at present is that BMP receptor signaling, mediated by BMP2/4/6/7 and/or activin, initiates the effects of the traumatic insults that lead to HO. The hypothesis underlying our current studies, and our experimental findings, challenge this view. Although BMP receptor signaling clearly plays a central role in HO, we find that BMP signaling is a later rather than early part of the signaling cascade that culminates in HO. Among over a dozen BMP-overexpressing transgenic lines, the Nse-BMP4 line utilized in the current studies is the only one that recapitulates the hallmarks of both aHO and FOP.^11,22^ This reflects the characteristics of the Nse promoter that drives the transgene construct. In Nse-BMP4 mice, the Nse transgene is induced in Mϕ by injury, but the injury-induced expression of BMP4 does not become significant until 3 days p.i. (Supplementary Fig. 1). This suggests that increased BMP signaling is not the initiating event in Nse-BMP4 mice but rather a necessary factor for propagating the pathophysiologic process.

Previous studies have shown that, unlike normal skeletogenesis, both innate and adaptive immunities contribute to HO.^11^ In particular, Mϕ are both abundant and persistent in local HO lesions, and limiting the influx of monocytes/Mϕ reduces or prevents experimental HO.^11^ This suggests an essential early role for Mϕ in triggering HO. We found that both stimulatory and inhibitory ICs were expressed by Mϕ in HO lesions. In general, the percentage of cells expressing stimulatory ICs was markedly increased in the early stages but decreased at later stages of HO, whereas the percentage of cells expressing inhibitory ICs was more variable with some increased earlier and some later during the HO process. Interfering with the expression of inhibitory ICs resulted in a truly remarkable reduction in HO with almost total prevention of HO even 2 months post-injury in the Nse-BMP4 mice. Conversely, interfering with the expression of stimulatory ICs markedly increased the severity of the HO. These observations highlight the central role of ICs in the altered immune homeostasis that leads to HO.

The observation that immune suppressants such as Rapamycin and Ebselen suppress HO would seem to suggest that overactive immune responses are responsible for HO. However, the findings that loss of function of stimulatory ICs facilitated HO, whereas loss of function of inhibitory ICs inhibited HO would seem to suggest that depressed immune responses are responsible. What underlies these seemingly contradictory sets of observations? We found that the percentage of CD206+/F4/80+ Mϕ in lesion sites increased progressively with time. In fact, <1% of the Mϕ expressed CD206 at the time of injury while about 90% of the cells expressed CD206 within several weeks. Similarly, there was a conversion in the ICs expressed by the Mϕ from stimulatory to inhibitory ones. This suggests a dynamic biphasic process, i.e., overactive early and depressed later immune responses that both are necessary preconditions for the subsequent HO. Thus blocking either phase may be sufficient to prevent HO.

In earlier studies, we identified dysregulation of local stem/progenitor cells as a common cellular mechanism for HO,^11^ and many subsequent studies have characterized both the lineage of the stem cells and the cellular and molecular components of the stem cell niche that presdisposes them to osteogenic differentiation.^23,25,27–32^ Stem/progenitor cell differentiation is greatly influenced by the ECM in which the cells reside, and increased deposition of ECM proteins is a consistent feature of the early phases of HO.^33–37^ In view of the central role of Mϕ in HO,^38–41^ we questioned whether they may alter the ECM during the HO process. We found that expression by F4/80 Mϕ of both MMP12 and Adamts19 was reduced after injury in Nse-BMP4 mice, whereas MMP12 increased in WT mice, suggesting a possible mechanism for the increase in ECM proteins in the lesion.

Although it is always difficult to extrapolate from animal models of disease to humans, the cellular and molecular features of human HO are very similar to what is observed in Nse-BMP4 mice.^28^ Thus we hypothesize that a BMP-dependent, injury-induced stem cell niche is a common mechanism of HO^28^ and that the altered immune homeostasis observed in the animal model are part of the process in humans. Importantly, we specifically used FDA-approved IC inhibitors in these studies to enhance the potential translational implications. Our findings suggest that treatment with IC Abs, in particular those that target inhibitory ICs, may provide a therapeutic approach to this currently untreatable clinical problem.

## Materials and methods

### Animals and injury models

Nse-BMP4 transgenic mice, described previously,^11,12,22–24^ express BMP4 under the control of promoter of Nse. HO was induced by intramuscular injection of cardiotoxin (Sigma), according to previous reports.^11,12,23,24^ All animal experiments in this study were approved by the Animal Care and Use Committees at Anhui Medical University (Protocol: LLSC20140042) and Northwestern University (Protocol: IS00001002).

### Quantification of local immune cell infiltration after injury

Immunostaining for different markers were performed as previously described.^11,25^ Briefly, sections were pre-fixed with 4% paraformaldehyde in phosphate-buffered saline (PBS). Nonspecific binding was blocked with 10% normal serum diluted in 1% bovine serum albumin (BSA; Jackson ImmunoResearch Laboratories, West Grove, PA) and 0.25% Triton X-100 (Sigma) for 1 h at room temperature. The sections then were incubated with primary Abs diluted with 1% BSA+0.25% Triton X-100 at 4 °C overnight (Primary Abs used in this study are summarized in Supplementary Table 1.). After washing, the sections were incubated with appropriate secondary Abs (Alexa Fluor 488, Alexa Fluor 594 conjugated Abs, Thermo Fisher Scientific) diluted with 1% BSA+0.25% Triton X-100 in a dark at room temperature for 2 h and counterstained with 4, 6-diamidino-2-phenylindole (1:5 000). All fluorescent images were taken using ZEISS Axio Observer (Carl Zeiss, Germany). Signals from all channels were collected separately and overlaid in DPViewer.

### Functional modulation of immune responses with immunosuppressants or IC blockade (neutralizing Abs against ICs)

Nes-BMP4 mice (*n* = 4) were treated with Rapamycin (5 mg·kg^–1^) or Ebselen (1 mg·kg^–1^), every other day for 2 weeks (8 injections) through intraperitoneal (i.p.) injection starting 1 day p.i. Control Nse-BMP4 mice were treated with vehicle on the same schedule. Every other day, dosing was used to minimize trauma to skin and abdominal muscle so that HO was not triggered by the injections. For IC blockade, the mice were treated with specific neutralizing Abs against stimulatory ICs, including CD40L (BioxCell, at the dosages of 20, 100, and 500 μg per injection) and CD134L (BioxCell, at the dosages of 60, 120, and 300 μg per injection), and inhibitory ICs, including CTLA-4 (BioxCell, at the dosages of 10, 50, and 250 μg per injection), PD1 (BioxCell, at the dosages of 10, 50, and 250 μg per injection), and PD-L1 (BioxCell, at the dosages of 24, 120, and 600 μg per injection), through the tail vein every other day for 1 week (4 injections) starting 1 day p.i.

### X-ray and micro-CT imaging

For X-ray, mice were anesthetized by 1% pentobarbital (150 μL i.p.) and the images of radio-opaque HO were acquired by whole-body X-ray examination at 38 kv, 28 mA, 30 s (Bruker, USA). To quantitatively measure HO volume, micro-CT (PerkinElmer, USA) was used with the setting parameters of 180° rotation, constant 90 kv voltage, and voxel size 72 μm, and the 3D images were reconstructed by the software package of the system.

### Western blotting

Lesional tissues or isolated Mϕ were lysed with RIPA buffer (Beyotime Biotechnology, China). Protein concentration was assessed by Bradford assay (Bio-Rad laboratories, USA). Protein samples (20 μg) were resolved using 8% polyacrylamide gel and electrophoretically transferred to nitrocellulose and then blocked with non-fat milk in 0.1% Tween-20 in PBS for 1 h. Membranes were then incubated with primary Abs (Supplementary Table 1) at room temperature for 1.5 h, and after washing, the membranes were incubated with horseradish peroxidase-conjugated secondary Abs. The specific signals were detected using the enhanced chemiluminescence western blot detection system (Odyssey, USA) after washing, following the manufacturer’s instructions. β-Actin was used as the loading control.

### RNA extraction and qRT-PCR analysis

An MirVana miRNA Kit (Takara, China) was used to extract total RNA from lesional tissues and Mϕ following the manufacturer’s instructions. PrimeScriptRT Reagent Kit (Takara, China) was used to synthesize the first-strand cDNA. Expression of various genes was quantified by the Real-time PCR Mixture assays (Takara, China). β-Actin was used as the internal control. All of the primer sequences and product sizes are summarized in Supplementary Table 2.

### Study of lesional Mϕ

For tissue Mϕ, F4/80+ Mϕ were sorted from injured tissues at different times through a MACS Kit (Miltenyi, USA), according to the manufacturer’s instruction, and the F4/80+ Mϕ were further analyzed by flow cytometry (BD, USA), using CD206 Ab (BD Pharmingen, USA) diluted with flow cytometry buffer (PBS+0.5%BSA+0.09% Sodium Azide). Flow cytometric data were analyzed by the FlowJo software (Tree Star, Inc).

### Statistical analyses

Data are reported as means ± standard deviation. Statistical analyses between two groups were performed using Student’s *t* test via SPSS 16.0 (SPSS Science, Chicago, IL). Statistical analyses between multiple groups were performed using one-way analysis of variance (ANOVA) or ANOVA with repeated measures followed by Bonferroni’s post hoc test. *P* < 0.05 was considered as statistically significant.

## Supplementary information

Supplementary figure 1

Supplementary figure 2

Suppementary Figure 3

Supplementary Tables

## Data Availability

All data associated with this study are available in the main text or the Supplementary Materials. All data and materials used in the analysis are available to any researcher for purposes of reproducing or extending the analysis.
