# Supplementary figures and images for: Inhibition of immune checkpoints prevents injury-induced heterotopic ossification
Source: Bone Res. 2019 Nov 1;7:33. doi: 10.1038/s41413-019-0074-7 (PMC6823457; doi:10.1038/s41413-019-0074-7)

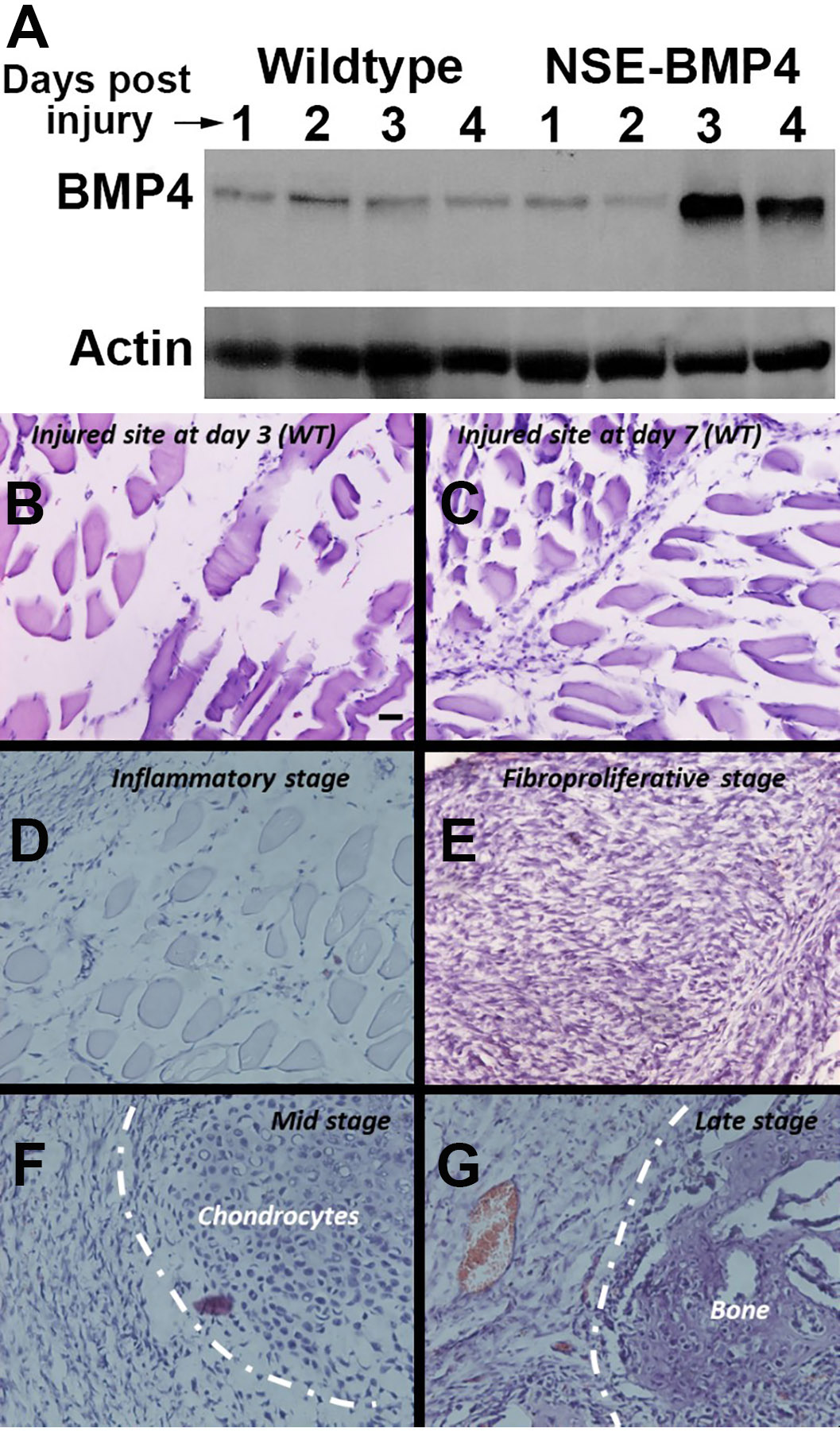

Supplement: Supplementary file 1 — Supplementary figure 1 [file 41413_2019_74_MOESM1_ESM.tif]

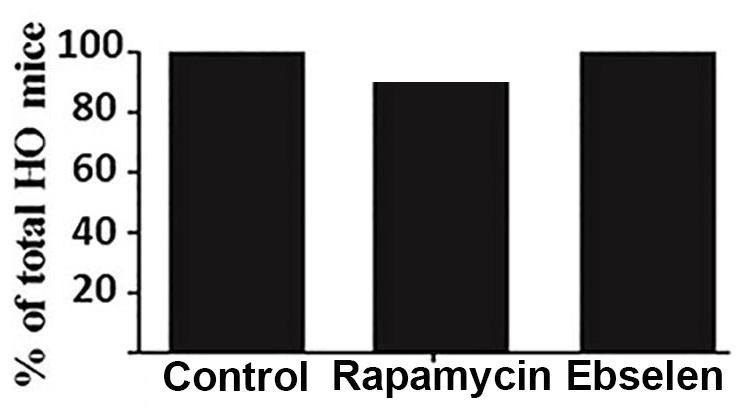

Supplement: Supplementary file 2 — Supplementary figure 2 [file 41413_2019_74_MOESM2_ESM.tif]

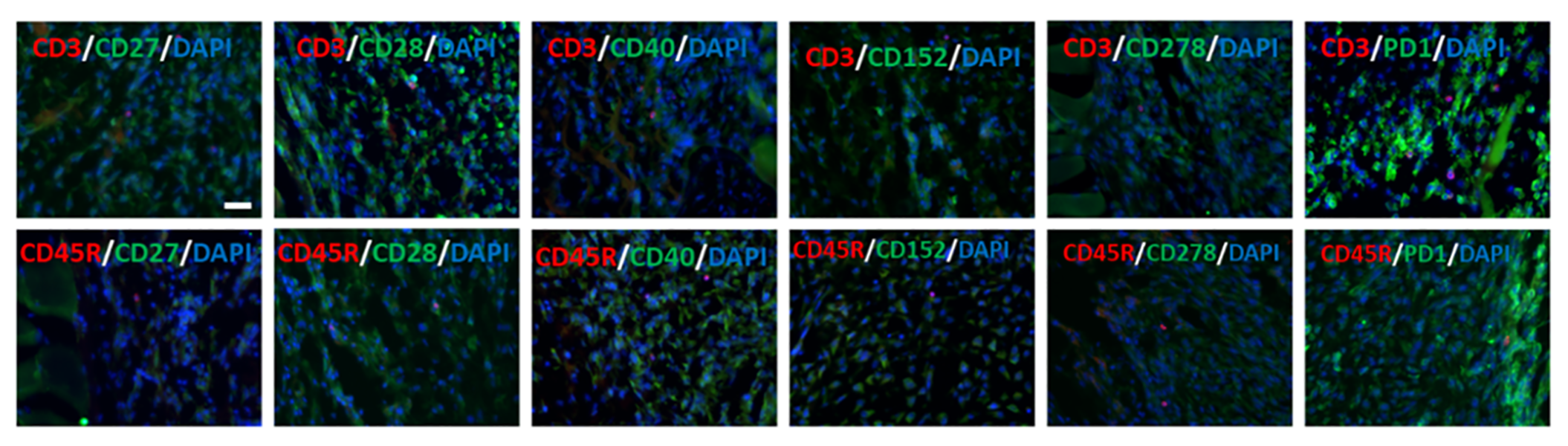

Supplement: Supplementary file 3 — Suppementary Figure 3 [file 41413_2019_74_MOESM3_ESM.tif]
