# Supplementary material for: Inhibition of immune checkpoints prevents injury-induced heterotopic ossification
Source: Bone Res. 2019 Nov 1;7:33. doi: 10.1038/s41413-019-0074-7 (PMC6823457; doi:10.1038/s41413-019-0074-7)

**Supplemental figure 1**

**Expression of BMP4 and histological features of lesional tissues of WT and Nse-BMP4 mice.** A) Western analysis for BMP4 expression at the lesion site in WT and NSE-BMP4 mice. There are no differences in expression for the first two days after injury, but a large increase occurred on day 3 in NSE-BMP4 mice. B-C) H&E staining showing the early morphological features of injured muscles at day 3 & 7 p.i. in WT mice, D-G) H&E staining showed the typical morphological features of HO from the inflammatory stage to the fibroproliferative stage (1 week after injury), the condensation and chondrogenesis stage (2 weeks after injury) and finally endochondral bone formation injury (4 weeks after injury).

**Supplemental Figure 2. Ebselen and Rapamycin fail to block HO when treatment is delayed until day 10.** Treatment with Ebselen or Rapamycin was delayed until day 10 and then given in the same dosages as in Figure 1. Nine of ten mice treated with Rapamycin developed HO and all 10 mice treated with Ebselen developed HO.

**Supplemental Figure 3. Immune checkpoint proteins were rarely co-localized within lymphocytes** Contrary to our original expectation, immune checkpoint proteins were rarely co-localized within lymphocytes in lesional tissues of HO. Top panels:  Immunofluorescence staining showing the co-localization of CD3 and ICs at 2W p.i.. Bottom panels: Immunofluorescence staining showing the co-localization of CD45R and ICs at 2W p.i..

**Suppl. Table 1 Summary of Primary Antibodies Used in this Study**
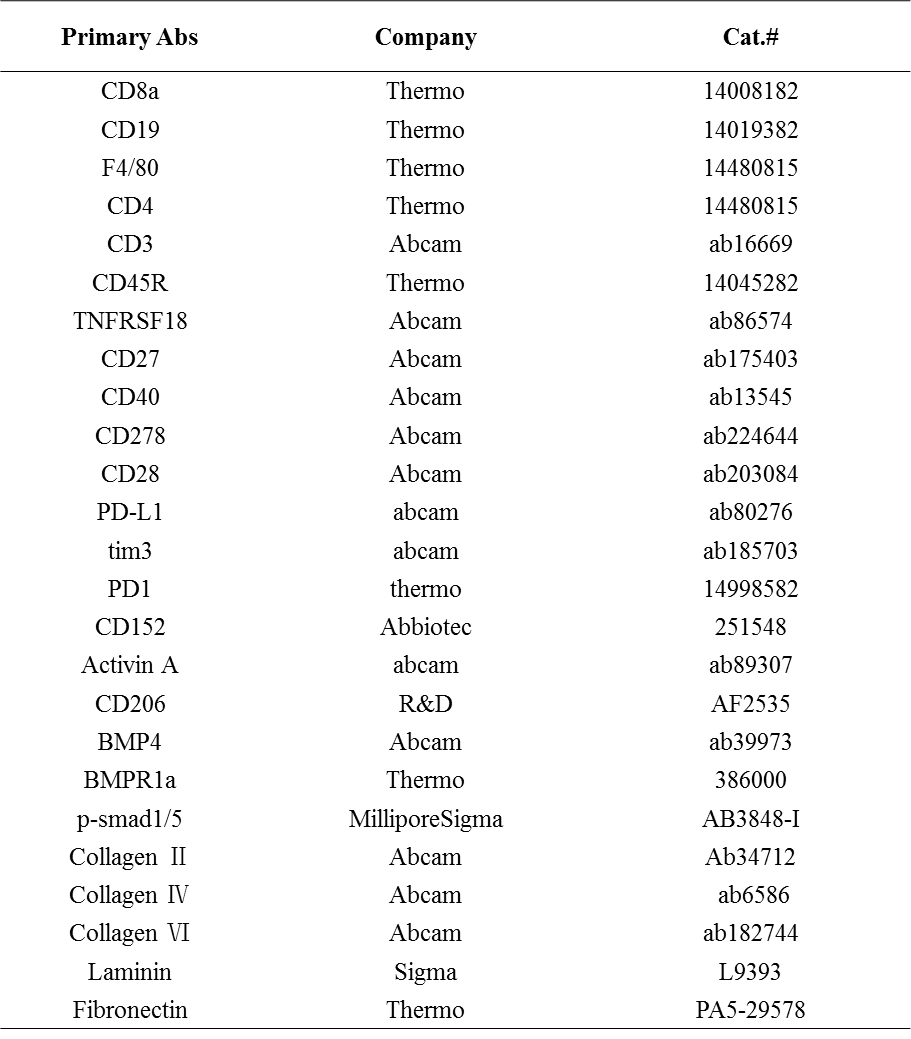


**Suppl. Table 2: Summary of qRT-PCR Primers Used in this Study**


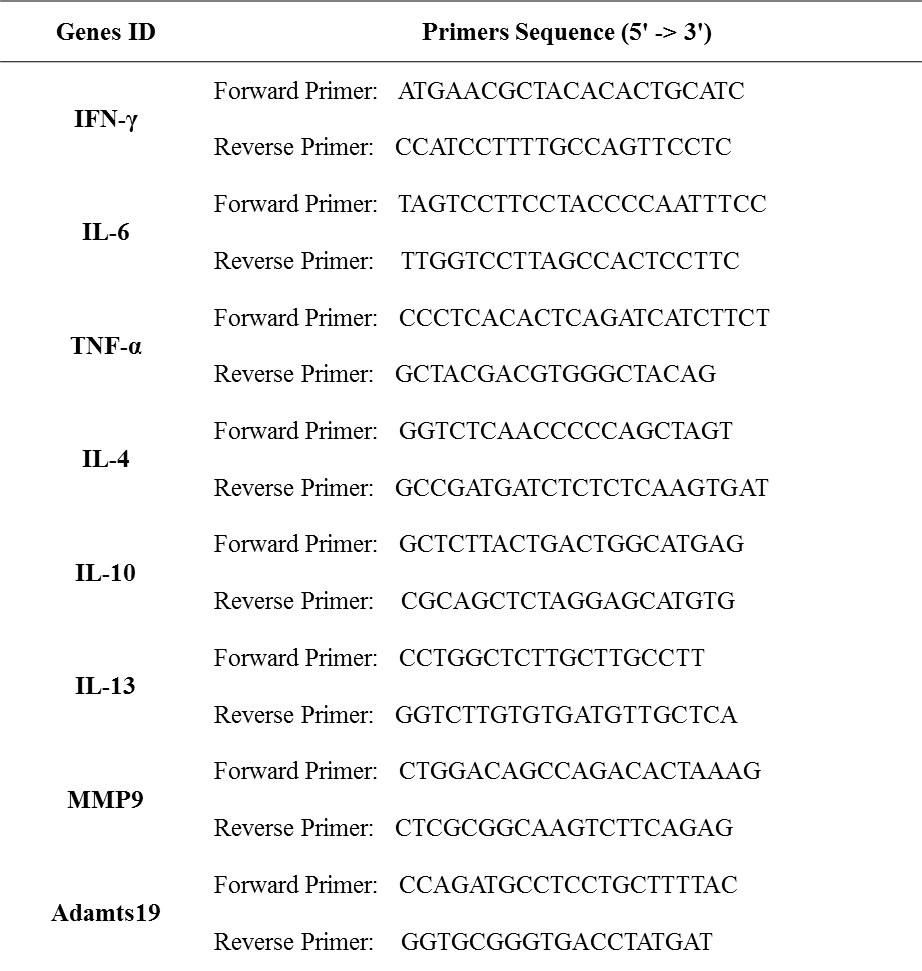

Supplement: Supplementary file 4 — Supplementary Tables [file 41413_2019_74_MOESM4_ESM.docx]
